# Machine learning-based modeling of acute respiratory failure following emergency general surgery operations

**DOI:** 10.1371/journal.pone.0267733

**Published:** 2022-04-28

**Authors:** Joseph Hadaya, Arjun Verma, Yas Sanaiha, Ramin Ramezani, Nida Qadir, Peyman Benharash

**Affiliations:** 1 Cardiovascular Outcomes Research Laboratories, Division of Cardiac Surgery, David Geffen School of Medicine, University of California, Los Angeles, California, United States of America; 2 Department of Computer Science, University of California, Los Angeles, California, United States of America; 3 Division of Pulmonary and Critical Care Medicine, David Geffen School of Medicine, University of California, Los Angeles, California, United States of America; Ospedale Sant’Antonio, ITALY

## Abstract

**Background:**

Emergency general surgery (EGS) operations are associated with substantial risk of morbidity including postoperative respiratory failure (PRF). While existing risk models are not widely utilized and rely on traditional statistical methods, application of machine learning (ML) in prediction of PRF following EGS remains unexplored.

**Objective:**

The present study aimed to develop ML-based prediction models for respiratory failure following EGS and compare their performance to traditional regression models using a nationally-representative cohort.

**Methods:**

Non-elective hospitalizations for EGS (appendectomy, cholecystectomy, repair of perforated ulcer, large or small bowel resection, lysis of adhesions) were identified in the 2016–18 Nationwide Readmissions Database. Factors associated with PRF were identified using ML techniques and logistic regression. The performance of XGBoost and logistic regression was evaluated using the receiver operating characteristic curve and coefficient of determination (R^2^). The impact of PRF on mortality, length of stay (LOS) and hospitalization costs was secondarily assessed using generalized linear models.

**Results:**

Of 1,003,703 hospitalizations, 8.8% developed PRF. The XGBoost model exhibited slightly superior discrimination compared to logistic regression (0.900, 95% CI 0.899–0.901 vs 0.894, 95% CI 0.862–0.896). Compared to logistic regression, XGBoost demonstrated excellent calibration across all risk levels (R^2^: 0.998 vs 0.962). Congestive heart failure, neurologic disorders, and coagulopathy were significantly associated with increased risk of PRF. After risk-adjustment, PRF was associated with 10-fold greater odds (95% confidence interval (CI) 9.8–11.1) of mortality and incremental increases in LOS by 3.1 days (95% CI 3.0–3.2) and $11,900 (95% CI 11,600–12,300) in costs.

**Conclusions:**

Logistic regression and XGBoost perform similarly in overall classification of PRF risk. However, due to superior calibration at extremes of risk, ML-based models may prove more useful in the clinical setting, where probabilities rather than classifications are desired.

## Introduction

Postoperative respiratory failure (PRF) occurs in 1–7% of patients undergoing non-cardiac surgery and is associated with substantial risk of mortality as well as excess and unanticipated healthcare expenditures [1–4]. Rates of PRF are considered metrics for quality of care by several organizations including the Agency for Healthcare Research Quality and National Quality Forum [5,6]. Importantly, PRF is a component of composite claims-based patient safety measures tracked by the Centers for Medicare and Medicaid Services, and may impact reimbursement in pay-for-performance schemes [7]. While the pathophysiology of PRF is complex and not completely understood, patients requiring emergency operations are at particularly high risk for this complication [8]. In fact, American Society of Anesthesiologists (ASA) class and type of operation are strongly factored into a PRF risk calculator developed by Gupta et al. nearly a decade ago using the National Surgical Quality Improvement Program (NSQIP) data files [9].

Emergency general surgery (EGS) operations, ranging from cholecystectomy to large bowel resection, are among the most common inpatient operations in the United States and are associated with significant morbidity, with reported rates of respiratory complications as high as 25 to 47% [10,11]. The risk of PRF may be particularly high in patients undergoing EGS due to the acuity of presentation, physiologic derangements and systemic inflammation, as well as altered abdominal wall dynamics. Respiratory complications, particularly PRF, have been consistently associated with higher rates of mortality in patients undergoing EGS operations [8,10,11].

*A priori* prediction of postoperative events including PRF and mortality are paramount to value-based healthcare delivery, shared decision making, and development of targeted quality improvement efforts. Appropriate selection of alternatives to surgical intervention, such as cholecystostomy tubes in lieu of cholecystectomy for cholecystitis in high-risk patients, also rely on the implicit risk of complications and death. In a review of available prediction tools for pulmonary complications, Nijbroek et al. evaluated 19 models and found poor external validation in nearly all cases [12]. Similarly, several of these prediction models, such as those derived from NSQIP, rely on data from generally high-performing hospitals and may fail to capture the pragmatic incidence and risk factors associated with PRF [13].

In the present study, we used nationally representative data derived from over 16 million annual hospitalizations to develop predictive models for PRF and assessed its impact on select clinical and financial endpoints. We compared the performance of machine learning models to traditional logistic regression, evaluating the calibration, discrimination and out-of-sample validity of each of these approaches. We hypothesized improved performance of machine learning over logistic methods.

## Methods

### Data source and study population

The 2016–2018 Nationwide Readmissions Database (NRD) was used to identify relevant patient and hospital level information. Maintained as part of the Healthcare Costs and Utilization Project, the NRD is an all-payer administrative database that provides accurate estimates for more than 17 million discharges representing 56% of annual hospitalizations in the United States [14]. Appropriate national estimates are obtained using hospital discharge weights provided by the NRD. Due to the deidentified nature of the NRD, the present study was deemed exempt from full review by the University of California, Los Angeles Institutional Review Board.

All adult (≥18 years of age) hospitalizations with procedural codes for any of six emergency general surgery (EGS) procedures during index hospitalization were identified. Operations comprising EGS including large or small bowel resection, cholecystectomy, repair of perforated ulcer, lysis of adhesions and appendectomy were identified using *International Classification of Diseases*, *Tenth Revision* (ICD-10) procedure codes, as previously described (S1 Table) [15]. These procedures were selected due to their frequency and relevance to clinical practice across all hospital types, and capture approximately 80% of the national burden of EGS [16]. Elective or trauma-associated hospitalizations as well as those involving transfers from other inpatient facilities were excluded. In addition, records with missing data for mortality, age and sex were excluded (<1%). To maintain a consistent definition of EGS operations, as well as reduce the risk of capturing preoperative respiratory failure in our cohort, only patients undergoing an operation within 2 days of index admission were considered in our study [16].

### Variable and outcome definitions

The primary study outcome was acute respiratory failure, defined using ICD-10 diagnosis codes (S1 Table). Baseline characteristics, including age, sex, income quartile and insurance status, were defined in accordance with the NRD data dictionary [17]. Comorbidities were ascertained using ICD-10 diagnosis codes or according to available fields in NRD. The Elixhauser Comorbidity Index, a previously validated aggregate score of 30 chronic conditions was utilized to quantify the burden of chronic illness [18]. Hospital level variables were defined according to the NRD and included teaching status and bed size [17].

Normally distributed variables are reported as mean and standard deviation (SD), while those with skewed distributions are summarized using median and interquartile range (IQR). Continuous variables were compared using the adjusted Wald or Mann-Whitney U test, as appropriate. Categorical variables are reported as proportions (%) and were compared using the Pearson’s chi square test. Total hospitalization costs were generated by application of hospital specific cost-to-charge ratios and inflation adjusted to 2018 [19]. Generalized linear models were used to evaluate the impact of PRF on mortality, length of stay (LOS), hospitalization costs, and non-home discharge. A gamma error distribution with log-link function was used for costs, and Gaussian distribution with square root-link used for length of stay. Covariates for adjusted analysis were selected autonomously using elastic net regularization, a technique which implements the L1 and L2 penalties to reduce collinearity and optimize model generalizability. Regression outcomes are reported as adjusted odds ratios for discrete and β coefficients for continuous variables, with 95% confidence interval (CI). Statistical significance was set at *P* < 0.05.

### Predictive modeling techniques

Machine learning (ML) is a branch of data analytics that adopts an automated approach to predictive model development. It has previously been demonstrated that ML models exhibit superior discrimination and predictive power for several clinical applications compared to traditional linear methodology [20–22]. Specifically, decision tree based ML models readily capture complex, non-linear relationships between covariates and outcomes of interest.

We compared the performance of eXtreme Gradient Boosting (XGBoost) to traditional logistic regression (LR). XGBoost is an ensemble model in which a multitude (hundreds to thousands) of weakly predictive decision trees are trained in a stage-wise fashion [23]. By correcting for errors from previous iterations, the model is refined and performance is improved as subsequent decision trees are trained. The final output of the model is the most commonly predicted class from each individual decision tree. Furthermore, XGBoost is a computationally efficient implementation of gradient boosting and dramatically reduces training time by distributing tasks across multiple central processing units [23]. The performance of machine learning algorithms can be optimized through the selection of appropriate hyperparameters, which are used to control the learning process. To obtain the greatest performance, hyperparameter tuning was conducted using a randomized search to maximize the area under the receiver operating characteristic (AUC). We used 500 estimators with a maximum tree depth of 2 and an L1 regularization value of 1.

### Model development strategy

We developed 4 distinct models to predict PRF–*XGBoost complete*, *LR complete*, *XGBoost sparse*, *LR sparse*. The *complete* models contain the maximum number of covariates necessary to yield the highest predictive performance. In contrast, the *sparse* models had a limited number of input features and were designed to be more portable for use in the clinical setting.

**Fig 1:**
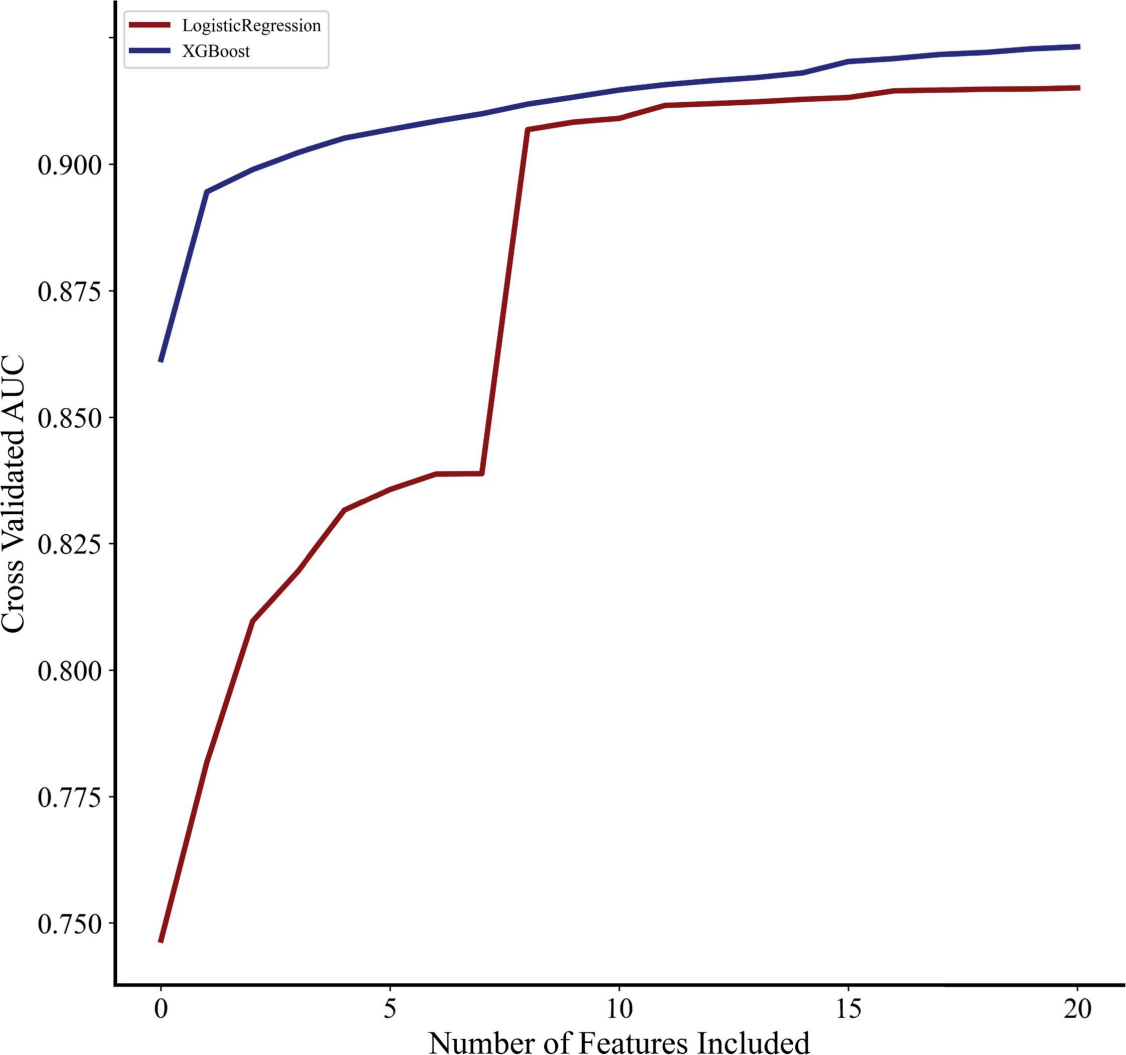
Relationship between covariates and AUC. Impact of number of model covariates on area under the receiver operating characteristic (AUC).

Only preoperative characteristics and operative type were considered for inclusion as covariates in all predictive models (S2 Table). Covariates in the *complete* LR and XGBoost models were chosen by applying elastic net regularization [24,25]. To develop the *sparse* models, recursive feature elimination (RFE) was used to identify the fewest number of covariates needed to adequately predict PRF [26]. Briefly, this technique exhaustively trains a multitude of predictive models using all possible combinations of covariates and subsequently evaluates the AUC. Subsequently, the number of covariates and AUC were plotted to determine the minimum number of covariates needed to demonstrate acceptable discrimination. As shown in Fig 1, after the inclusion of 9 covariates, AUC for both logistic regression and XGBoost did not increase significantly. Therefore, the *sparse* model was developed with only 9 input features which most strongly predicted PRF, as identified by RFE. The entire study cohort was randomly split into training (75%) and testing (25%) datasets. Models were derived using known data (training) and evaluated using testing data. This process was repeated 10 times to generate cross-validated performance metrics.

### Model comparison and interpretation

Model discrimination was compared using out-of-sample receiver operating characteristics curves (AUC). In addition, recall (sensitivity), precision (positive predictive value) and balanced accuracy were used to evaluate classification performance. Precision-recall curves were constructed to show sensitivity and positive predictive value across all risk-thresholds, while the mean average precision (mAP) was calculated as the area under the precision-recall curve. Calibration curves were constructed by plotting observed versus expected PRF rates. Model calibration was quantified using the slope and intercept of the best fit line, as well as the coefficient of determination (R^2^) to between observed and predicted. In addition, the Brier score was used to measure the accuracy of probabilistic predictions. Covariates were ranked by feature importance, which was calculated as the average increase in predictive performance across all decision trees attributable to the inclusion of each variable. Feature importance values of each covariate were retrieved from the XGBoost model using the *feature_importance* attribute. Statistical analysis was conducted using Stata 16.0 (StataCorp, College Station, TX) and Python version 3.9 (Python Software Foundation, Wilmington, DA). The *xgboost* and *sklearn* packages of Python were used to develop predictive models as described above [23,27]. The Python code to develop and evaluate our ML models has been previously published by our group [28].

## Results

### Baseline demographics and outcomes

Of an estimated 1,003,703 patients with EGS related admissions during the study period, 8.8% developed PRF. Patients with PRF were on average older, less commonly female and had a higher burden of chronic conditions as measured by the Elixhauser comorbidity index, compared to others (Table 1). On crude analysis, patients with PRF had significantly higher rates of in-hospital mortality and non-home discharge (Table 1). In addition, LOS and hospitalization costs were greater among those who developed PRF. Upon adjusted analysis, PRF was associated greater odds of mortality (10.1, 95% CI 9.8–11.1) and non-home discharge (3.5, 95% CI 3.4–3.6, Table 2). In addition, PRF was associated with a 3.1-day increment (95% CI 3.0–3.2) in LOS and $11,900 in total hospitalization costs (95% CI 11,600–12,300, Table 2).

**Table 1:** Comparison of patient and hospital characteristics stratified by the development of postoperative respiratory failure (PRF).

	No PRF (n = 1,003,703)	PRF (n = 88,308)	*P* value
Female (%)	60.3	52.1	<0.001
Age (years)	55.0±18.4	68.4±14.0	<0.001
Primary Insurer (%)			<0.001
Private	36.8	17.3	
Medicare	36.7	68.4	
Medicaid	17.2	9.4	
Other Payer*	9.2	4.9	
Income Quartile (%)			<0.001
Fourth (Highest)	19.0	17.0	
Third	25.0	24.1	
Second	27.9	29.2	
First (Lowest)	28.2	29.8	
Operation (%)			<0.001
Large Bowel Resection	15.3	43.9	
Small Bowel Resection	8.7	21.3	
Cholecystectomy	62.1	18.1	
Repair of Perforated Ulcer	1.6	7.0	
Lysis of Adhesions	7.4	6.9	
Appendectomy	5.0	2.8	
Elixhauser Comorbidity Index	2.2±1.9	5.1±2.2	<0.001
Medical Conditions (%)			
Arrhythmia	12.7	40.5	<0.001
Chronic Liver Disease	7.3	13.3	<0.001
Chronic Lung Disorder	13.8	31.5	<0.001
Coagulopathy	3.5	21.0	<0.001
Congestive Heart Failure	5.1	26.4	<0.001
Coronary Artery Disease	10.3	23.6	<0.001
End Stage Renal Disease	1.3	4.7	<0.001
Hypothyroidism	10.4	14.3	<0.001
Malignancy	7.5	14.6	<0.001
Neurologic Disorder	3.7	22.0	<0.001
Valve Disorder	2.9	6.9	<0.001
Hospital Bed Size (%)			<0.001
Large	53.3	56.3	
Medium	29.1	28.6	
Small	17.6	15.0	
Teaching Hospital (%)	63.3	66.0	<0.001

Categorical variables reported as frequency and continuous as mean and standard deviation.

*Other payer includes uninsured and self-pay.

**Table 2:** Unadjusted and adjusted outcomes stratified by presence of postoperative respiratory failure (PRF).

Outcome	No PRF (n = 1,003,703)	PRF (n = 88,308)	*P* value	Estimate (Odds Ratio or β coefficient)	*P* value
Mortality	1.0	22.0	<0.001	10.4 (9.8–11.1)	<0.001
Non-home Discharge	7.2	48.2	<0.001	3.5 (3.4–3.6)	<0.001
Length of Stay (days)	3 (2–6)	11 (7–19)	<0.001	3.1 (3.0–3.2)	<0.001
Hospitalization Costs ($1,000)	13.1 (9.4–19.4)	39.1 (24.8–65.3)	<0.001	11.9 (11.6–12.3)	<0.001

Unadjusted outcomes reported as incidence per 100 (mortality and non-home discharge), or median and interquartile range (length of stay and costs). Adjusted outcomes reported as odds ratios or β coefficient for PRF vs. no PRF with 95% confidence intervals.

### Influence of variable selection on model performance

As exhibited in Fig 1, the XGBoost model consistently demonstrated superior discrimination with fewer covariates, compared to logistic regression. The most significant difference between XGBoost and LR was noted before the inclusion of 8 features (AUC 0.884 vs 0.812). However, for both models, maximum discrimination was observed after inclusion of all covariates.

### Model discrimination

**Fig 2:**
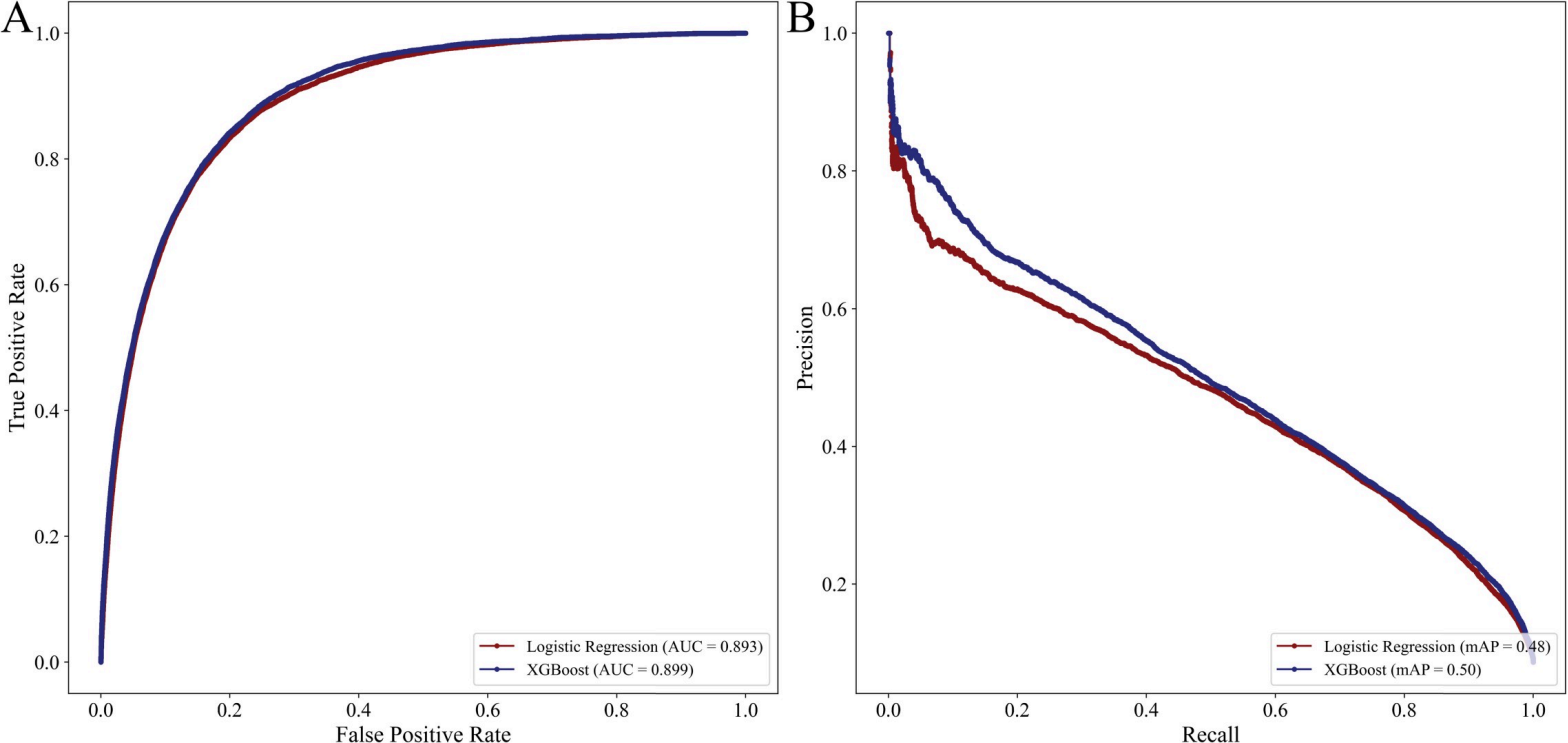
Receiver operating characteristics (2A) and precision recall curves (2B) for logistic regression and XGBoost with complete set of covariates.

**Fig 3:**
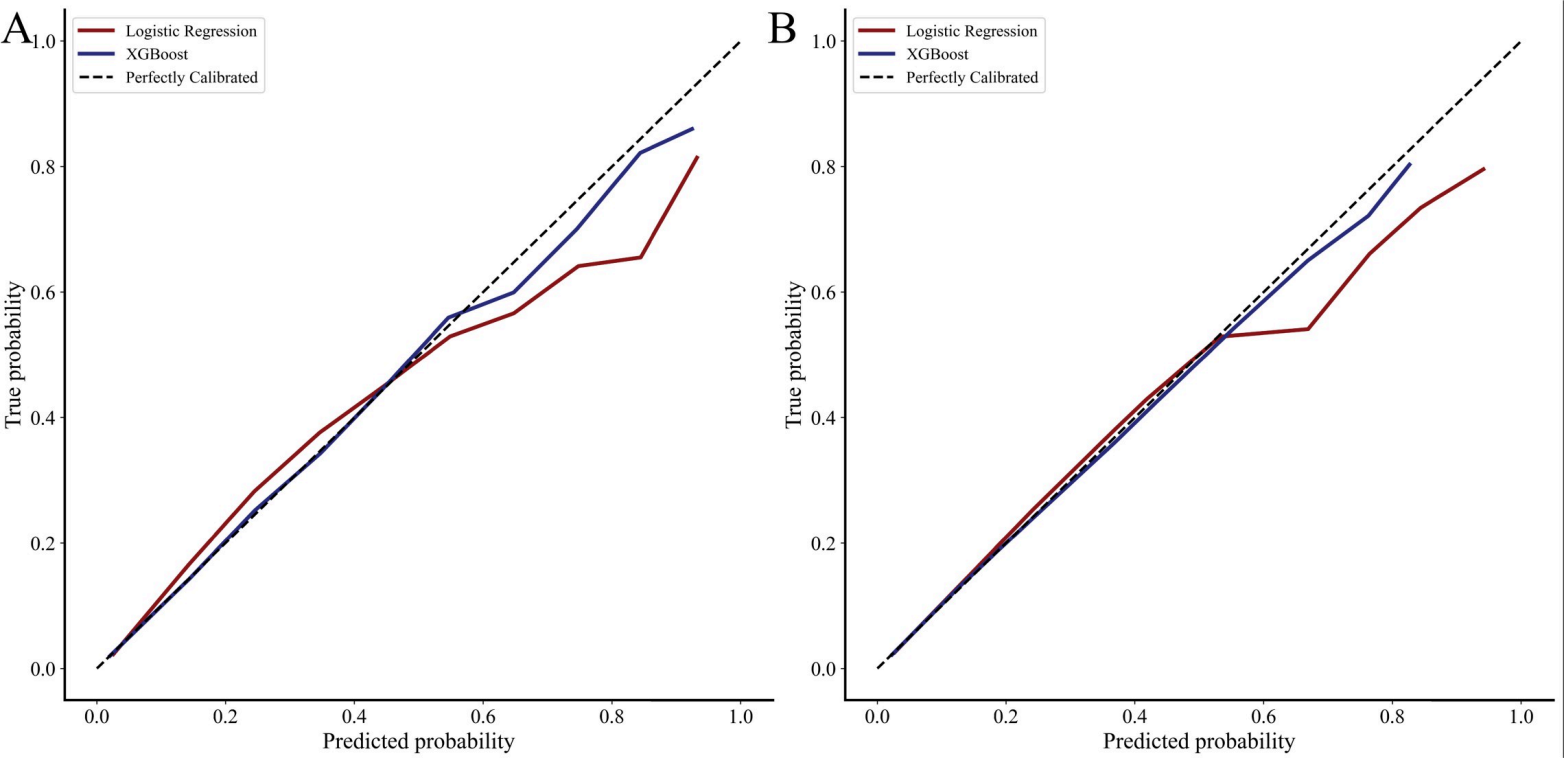
Calibration curves for logistic regression and XGBoost for complete (3A) and sparse (3B) feature sets. Complete set refers to the inclusion of all covariates in model development, while sparse set refers to the inclusion of 3 comorbidities (congestive heart failure, neurologic disorder, coagulopathy) and 6 emergency general surgery operative categories.

While the LR *complete* model had good discrimination in the validation cohort, the XGBoost *complete* model exhibited slightly greater AUC (0.900, 95% CI 0.899–0.901 vs 0.894, 95% CI 0.892–0.896, Fig 2). Despite similar AUC, the XGBoost *complete* classifier exhibited modestly increased recall compared to LR (0.270, 95% CI 0.268–0.272 vs 0.265, 95% CI 0.261–0.269). Additionally, the mAP of XGBoost *complete* was greater than LR (0.50 vs 0.48, Fig 2). In addition, both models had similar precision and balanced accuracy (Table 3). The LR *complete model* exhibited acceptable calibration, with increasing error at the high extremes of risk. However, the XGBoost *complete* model demonstrated excellent calibration, with a slope of 0.952, intercept of 0.02 and coefficient of determination of 0.993. Calibration plots for both predictive models are shown in Fig 3A.

**Table 3:** Performance metrics for logistic regression and machine learning based models.

	Complete Set		Sparse Set	
Metric	LR (95% CI)	XGBoost (95% CI)	LR (95% CI)	XGBoost (95% CI)
AUC	0.894 (0.892–0.896)	0.900 (0.899–0.901)	0.836 (0.833–0.839)	0.836 (0.833–0.839)
Recall	0.265 (0.261–0.269)	0.270 (0.268–0.272)	0.154 (0.151–0.157)	0.152 (0.150–0.154)
Precision	0.603 (0.597–0.609)	0.636 (0.631–0.641)	0.646 (0.637–0.655)	0.651 (0.644–0.658)
Balanced Accuracy	0.624 (0.622–0.626)	0.628 (0.627–0.629)	0.572 (0.571–0.573)	0.572 (0.571–0.573)
Brier Score	0.058 (0.057–0.059)	0.057 (0.056–0.058)	0.063 (0.062–0.064)	0.063 (0.062–0.064)

Metrics reported as mean with 95% confidence intervals (95% CI) and obtained via 10-fold cross validation. Complete set refers to the inclusion of all covariates in model development, while sparse set refers to the inclusion of 3 comorbidities (congestive heart failure, neurologic disorder, coagulopathy) and 6 emergency general surgery operative categories. LR, Logistic regression; AUC, Area under the receiver operating characteristic curve; XGBoost, Extreme gradient boosting.

Two additional models were developed to compare the performance of logistic regression and XGBoost with a sparse set of covariates. We considered the inclusion of 3 comorbidities (congestive heart failure, neurologic disorder, coagulopathy) and all 6 operative categories to be sparse. Consequently, both predictive models retained excellent discrimination (LR *sparse*: 0.836, 95% CI 0.833–0.839 vs XGBoost *sparse*: 0.836, 95% CI 0.833–0.839). However, the *sparse* XGBoost retained superior calibration with a slope of 0.975 vs 0.862, intercept of 0.01 vs 0.04 and R^2^ of 0.998 vs 0.962 (Fig 3B).

### Feature importance

**Fig 4:**
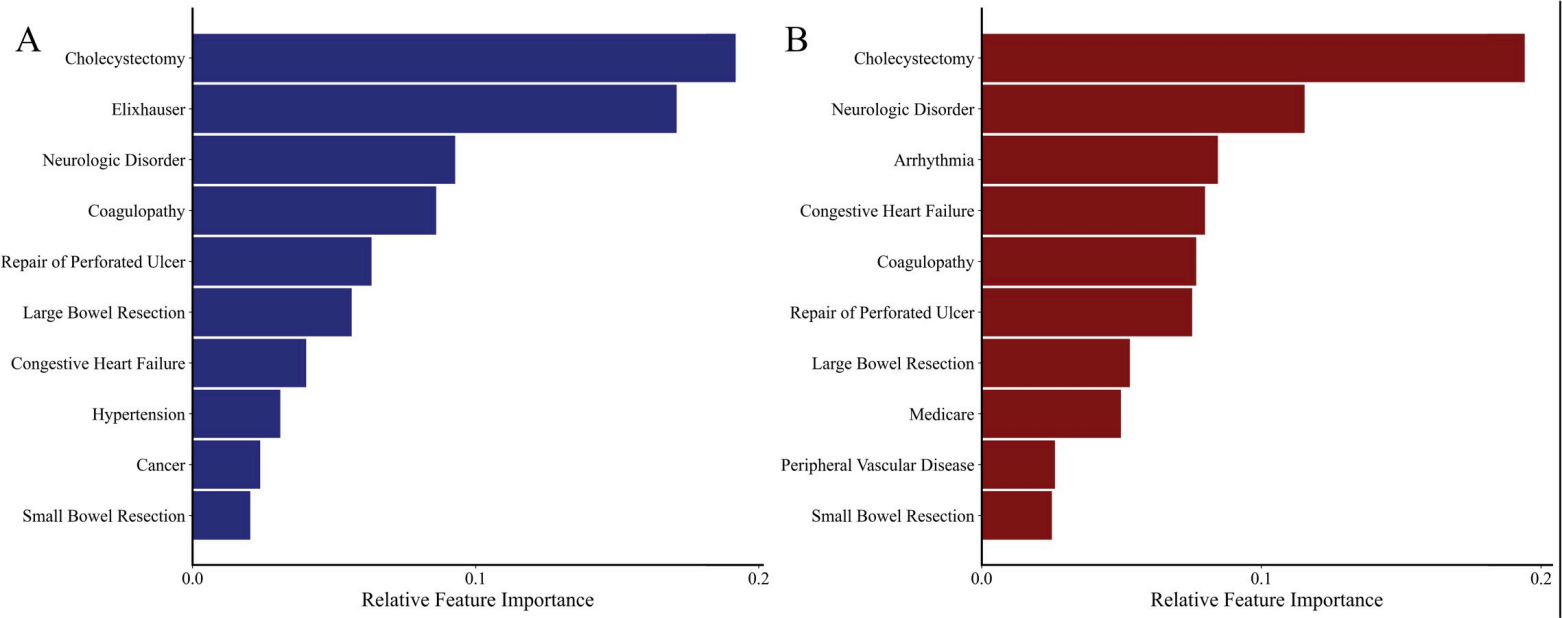
Feature importance of XGBoost model in predicting postoperative respiratory failure with (4A) and without (4B) the inclusion of the Elixhauser Comorbidity Index in models.

Covariates exhibiting a significant association with PRF are shown in Fig 4A and included operative type and Elixhauser Comorbidity Index (Fig 4A). Given that the Elixhauser Comorbidity Index is a composite of 30 comorbidities, we performed a subgroup analysis using the XGBoost algorithm to identify influential comorbidities by excluding this feature from the model. The most important features were congestive heart failure, neurologic disorders, coagulopathy and arrhythmia (Fig 4B).

## Discussion

Emergency general surgery operations are used to treat a heterogeneous group of patients with acute presentation and physiologic derangements. While several groups have previously reported EGS patients to be at increased risk of several complications, few have focused on postoperative respiratory failure. In the present study, we found nearly 10% of EGS patients to develop PRF, a complication that was associated with nearly tenfold greater risk of mortality and index hospitalization costs. Given its detrimental impact, we developed a machine learning based model to predict PRF using preoperative characteristics and compared this model to traditional logistic regression. Compared to traditional logistic regression, machine learning models exhibited improved discrimination and calibration, particularly with inclusion of few covariates and at high levels of risk. Our findings have important clinical implications and deserve further discussion.

Given the increasing burden of EGS across US centers, many large hospitals have developed specialized teams to manage this complex set of patients, who often require resuscitation and critical care management [29–31]. Despite such efforts, EGS operations are associated with high rates of cardiovascular and pulmonary complications, including PRF, noted in 10% of hospitalizations in the present work. From a pathophysiologic perspective, significant systemic inflammation present in EGS patients may result in capillary leak, and pulmonary edema, predisposing this group to pneumonia and respiratory compromise [32]. In addition to preexisting cardiovascular and pulmonary comorbidities, ranging from 10–30% in this study, postoperative pain may further impair lung mechanics and increase the risk of PRF. In the present work, congestive heart failure and renal dysfunction, among other medical conditions, were strongly associated with PRF; knowledge and inclusion of these risk factors in models may guide perioperative management protocols, such as fluid resuscitation [4]. Perioperative strategies, such as the use of short-acting neuromuscular blockers rather than long-acting agents, as well as selective use of nasogastric decompression, may mitigate risk of PRF, and should be considered for those at higher risk [33,34]. Importantly, while patients who underwent laparotomy and bowel resection and PRF had greater risk of mortality, those undergoing more common operations such as cholecystectomy, also fared poorly after development of PRF. This finding underscores the importance of risk assessment for all patients undergoing EGS, rather than just high-risk operations. With increasing availability of alternative non-surgical therapeutic strategies, accurate estimation of procedural risk may inform decision-making when alternatives are available. Given the high incidence of PRF and associated mortality, our risk model may better inform operative strategies, aid in shared decision making, and facilitate initiation of preemptive therapies that may reduce risk of PRF.

Most existing risk models for PRF have faced low adoption owing to a host of factors [12]. Gupta et al. created a logistic-regression derived risk calculator for procedures ranging from spine surgery, aortic surgery, breast, and head/neck procedures, and found the model to have good discriminatory power [9]. However, the model has been criticized by the inclusion of a broad set of unrelated operations, and inclusion of the American Society of Anesthesiologists Class (ASA), a variable that is ascertained intraoperatively and thought to be subjective [35]. Other risk scores rely on acute physiological parameters such as laboratory values and vital signs that are only found in select datasets such as the NSQIP [35]. Thus, their application to coding based national data and in centers that do not participate in NSQIP, are limited. Moreover, the nonlinear relationship of risk factors with PRF make logistic models complex and require explicit interaction terms. In the present work, we used the largest all payer administrative database in conjunction with machine learning and logistic models to derive and compare parsimonious prediction models. To the extent that was possible, we avoided inclusion of postoperative variables that would be associated with PRF in order to enhance the utility of such models during the preoperative phase. While logistic models demonstrated receiver-operating characteristics that were similar to ML, they had poor calibration at extremes of risk. With the high PRF risk regime representing a group that would benefit most from non-traditional clinical approaches, prospective evaluation of the ML model presented in this work is warranted. From an operational standpoint, the ML model reached optimal performance with the inclusion of fewer variables compared to LR and may be more straightforward to use.

As hospital and insurers develop value-based reimbursement paradigms, prevention of complications and mitigation of their impact on clinical outcomes will become increasingly relevant [7,36]. In the present work, PRF resulted in an $17,400 increment in hospitalization cost and 5.3 hospitalization days. A coding-based risk score for PRF, such as the present ML-based score, can be readily incorporated into electronic medical records and provide a first step in improving the care of these patients. Such a tool may help better engage providers and increase awareness of this particular common complication. More so, action items, such as implementation of care paths in the form of standardized orders or through physician and nursing led education protocols, may be prompted by accurate risk assessment. Similar risk assessment tools, particularly for sepsis screening, derive risk from medical record data, and have been effective in prompting diagnostic testing and interventions, with some studies demonstrating a mortality benefit [37]. Given the prevalence of these 6 operations, encompassing approximately 80% of emergency general surgery cases, accurate assessment of risk factors and practices to reduce the risk of PRF and its consequences are relevant to all hospital types and warrant further study [16].

This study has several limitations related to its retrospective design and the structure of the Nationwide Readmissions Database. Although comprehensive data regarding diagnoses and procedures performed are available in NRD, limited laboratory or physiologic information is available for inclusion in risk models. While we were unable to compare our machine-learning models to published algorithms, we utilized logistic regression with traditional methods for covariate selection for comparison. We further tested validity of our models using 10-fold cross validation. Finally, while the NRD is an administrative database, it captures approximately 17 million discharges annually, and provides nationally-representative estimates for 56% of the US population.

In this national study of emergency general surgery patients, we found a nearly 10% incidence of postoperative respiratory failure. We found that an ICD-coding based machine-learning approach resulted in superior model performance, particularly for those at high-risk of PRF. Such approaches to risk assessment and stratification may ultimately contribute to improved care, and, given their simplicity, should be strongly considered for inclusion in medical-record based tools. Given the prevalence of EGS across all hospital types, and the strong association of PRF with mortality and resource use, assessment of risk may strongly inform peri-procedural care and postoperative management.

## Supporting information

S1 Table List of International Classification of Diseases, Tenth Revision codes used for cohort definition. (DOCX)

S2 Table List of candidate predictors and covariates selected for adjusted analysis. (DOCX)
