# Supplementary material for: Machine learning-based modeling of acute respiratory failure following emergency general surgery operations
Source: PLoS One. 2022 Apr 28;17(4):e0267733. doi: 10.1371/journal.pone.0267733 (PMC9049563; doi:10.1371/journal.pone.0267733)
Supplement: S1 Table — (DOCX) [file pone.0267733.s001.docx]

**S1 Table. List of *International Classification of Diseases, Tenth Revision* codes used for cohort definition.**

|  | **ICD-10 Codes** |
| --- | --- |
| Lysis of Adhesions | 0DN80, 0DN83, 0DN84, 0DN90, 0DN93, 0DN94, 0DNA0, 0DN3,0DN4, 0DNB0, 0DNB3, 0DNB4, 0DNC0, 0DNC3, 0DNC4, 0DNE0, 0DNE3, 0DNE4, 0DNF0, 0DNF3, 0DNF4, 0DNG0, 0DNG3, 0DNG4, 0DNH0, 0DNH3, 0DNH4, 0DNJ0, 0DNJ3, 0DNJ4, 0DNK0, 0DNK3, 0DNK4, 0DNL0, 0DNL3, 0DNL4, 0DNM0, 0DNM3, 0DNM4, 0DNN0, 0DNN3, 0DNN4, 0DNP0, 0DNP3, 0DNP4, 0DNQ0, 0DNQ3, 0DNQ4 ,0DNR0, 0DNR3, 0DNR4, 0DNU0, 0DNU3, 0DNU4, 0DNV0, 0DNV3, 0DNV4, 0DNW0, 0DNW3, 0DNW4 |
| Appendectomy | 0DBJ0,0DBJ3,0DBJ4, 0DTJ0, 0DTJ4 |
| Cholecystectomy | 0FB40, 0FB43, 0FB44, 0FT40, 0FT44 |
| Small Bowel Resection | 0DB80, 0DB83, 0DB84, 0DB90, 0DB93, 0DB94, 0DBA0, 0DBA3, 0DBA4, 0DBB0, 0DBB3, 0DBB4, 0DT80, 0DT84, 0DT90, 0DT94, 0DTA0, 0DTA4, 0DTB0, 0DTB4 |
| Large Bowel Resection | 0DBE0, 0DBE3, 0DBE4, 0DBF0, 0DBF3, 0DBF4, 0DBF0, 0DBF3, 0DBF4, 0DBG0, 0DBG3, 0DBG4, 0DBH0, 0DBH3, 0DBH4, 0DBK0, 0DBK3, 0DBK4, 0DBL0, 0DBL3, 0DBL4, 0DBM0, 0DBM3, 0DBM4, 0DBN0, 0DBN3, 0DBN4, 0DBP0, 0DBP3, 0DBP4, 0DBQ0, 0DBQ3, 0DBQ4, 0DBC0, 0DBC3, 0DBC4, 0DTE0, 0DTE4, 0DTF0, 0DTF4, 0DTG0, 0DTG4, 0DTH0, 0DTH4, 0DTK0, 0DTK4, 0DTL0, 0DTL4, 0DTM0, 0DTM4, 0DTN0, 0DTN4, 0DTP0, 0DTP4, 0DTQ0, 0DTQ4, 0DTC0, 0DTC4 |
| Repair of Perforated Ulcer | 0DQ70, 0DQ73, 0DQ74, 0DQ60,0DQ63,0DQ64, 0DQ90, 0DQ93, 0DQ94 |
| Respiratory Failure | J95821, J95822, J9600, J9601, J9602, J9620, J9621, J9622, J9690, J9691, J9692 |
