# Supplementary material for: Machine learning-based modeling of acute respiratory failure following emergency general surgery operations
Source: PLoS One. 2022 Apr 28;17(4):e0267733. doi: 10.1371/journal.pone.0267733 (PMC9049563; doi:10.1371/journal.pone.0267733)
Supplement: S2 Table — (DOCX) [file pone.0267733.s002.docx]

**S2 Table. List of candidate predictors and covariates selected for adjusted analysis.**

| **Candidate Predictor Variables** | **Selected for Adjusted Analysis** |
| --- | --- |
| Age | x |
| Female | x |
| Elixhauser Comorbidity Index | x |
| Congestive Heart Failure | x |
| Coronary Artery Disease | x |
| Arrhythmia | x |
| Valve Disorder | x |
| Pulmonary Circulation Disorder | x |
| Peripheral Vascular Disease | x |
| Limb Ischemia | x |
| Uncomplicated Hypertension | x |
| Complicated Hypertension | x |
| Paralysis | x |
| Neurologic Disorder | x |
| Chronic Lung Disorder | x |
| Uncomplicated Diabetes | x |
| Complicated Diabetes | x |
| Hypothyroidism | x |
| End Stage Renal Disease | x |
| Liver Disease | x |
| Peptic Ulcer Disease | x |
| AIDS | x |
| Lymphoma | x |
| Metastatic Cancer | x |
| Non-metastatic Cancer | x |
| Cancer | x |
| Rheumatoid Arthritis/Collagen Vascular Diseases | x |
| Coagulopathy | x |
| Teaching Hospital | x |
| Large Bowel Resection | x |
| Small Bowel Resection | x |
| Cholecystectomy | x |
| Perforated Ulcer Repair | x |
| Lysis of Adhesions | x |
| Appendectomy | x |
| Income 0th-25th Percentile | x |
| Income 26th-50th Percentile | x |
| Income 51st-75th Percentile | x |
| Income 76th-100th Percentile | x |
| Private Insurance | x |
| Medicaid | x |
| Medicare | x |
| Other Payer/Self Pay | x |
| Hospital Bed Size (small) | x |
| Hospital Bed Size (medium) | x |
| Hospital Bed Size (large) | x |
